# Cardiac Tamponade During Catheter Atrial Fibrillation Ablation: A Life-Threatening Complication

**DOI:** 10.7759/cureus.44989

**Published:** 2023-09-10

**Authors:** Zahid Khan

**Affiliations:** 1 Acute Medicine, Mid and South Essex NHS Foundation Trust, Southend on Sea, GBR; 2 Cardiology, Bart’s Heart Center, London, GBR; 3 Cardiology and General Medicine, Barking, Havering and Redbridge University Hospitals NHS Trust, London, GBR; 4 Cardiology, Royal Free Hospital, London, GBR

**Keywords:** rhythm versus rate control, atriclip, left atrial appendage perforation, emergency sternotomy, emergency pericardiocentesis, massive pericardial effusion, direct current cardioversion, cardiac catheter ablation, atrial flutter rapid ventricular response, atrial fibrillation management

## Abstract

Catheter ablation has become an important treatment strategy for the management of atrial fibrillation (AF) in symptomatic patients. Pulmonary vein isolation (PVI) is increasingly used to restore rhythm in patients with AF and flutter. The serious procedural complication rate has significantly reduced over time and most patients undergo PVI without any adverse events. We present the case of a 70-year-old man with symptomatic AF who underwent elective PVI that was complicated by large pericardial effusion from left atrial appendage (LAA) perforation resulting in cardiac tamponade requiring emergency pericardiocentesis followed by sternotomy to suture the LAA. The perforated LAA was sutured and the LAA was closed surgically through sternotomy by using AtriClip and a large amount of blood was evacuated achieving good cardiac output and hemodynamic stability. A surgical PVI was performed twice restoring normal sinus rhythm. The patient was discharged home, however, he returned to the hospital a few days later with atrial flutter with a rapid ventricular response. He underwent direct current cardioversion (DCCV) and remained in sinus rhythm during the rest of his admission. His bisoprolol was switched to Sotalol to maintain normal sinus rhythm and he was discharged home with outpatient follow-up.

## Introduction

Atrial fibrillation (AF) ablation is one of the substantial treatment options for rhythm control, especially among patients who have symptomatic AF that is refractory to pharmacological and/or electrical cardioversion or among patients who are intolerant to anti-arrhythmic medications [1]. Cardiac tamponade is a rare complication of catheter ablation for AF or pulmonary vein isolation (PVI) and the incidence of delayed cardiac tamponade (DCT) has been reported as 0.2% [2]. The effectiveness of cardiac catheter ablation in AF has been well-established recently; however, care needs to be taken to minimize the procedure-related complications [3]. A large study showed that AF ablation-related major complications occurred in 4.54%‐6% of patients and the mortality rate associated with AF ablation was 0.15% [3]. The most common cause of AF ablation-related death was cardiac tamponade reported to be 1%‐1.31% [3,4]. Patients who develop cardiac tamponade due to atrial appendance perforation require emergency pericardiocentesis and/or emergency surgical repair [5]. It is important to mention that the incidence of serious periprocedural complications such as left atrial appendage (LAA) perforation is rare [4].

The serious complications of pericardial effusion and cardiac tamponade can occur anytime during the catheter ablation procedure. This can result from the trans-septal puncture, extensive catheter manipulation, application of radiofrequency (RF) energy, or steam pops under an intense anticoagulation regimen and the incidence was reported between 1.0% and 2.0% in another study [6]. The presumed mechanism of cardiac tamponade was related to the right ventricular apical ablation catheter placement, PVI, trans-septal puncture, cavotricuspid isthmus (CTI) ablation, and left atrial (LA) linear ablation in 33 patients whereas the cause was not clear in 18 patients [6]. We present a case of pericardial tamponade secondary to LLA perforation during LA mapping.

## Case presentation

**Figure 1:**
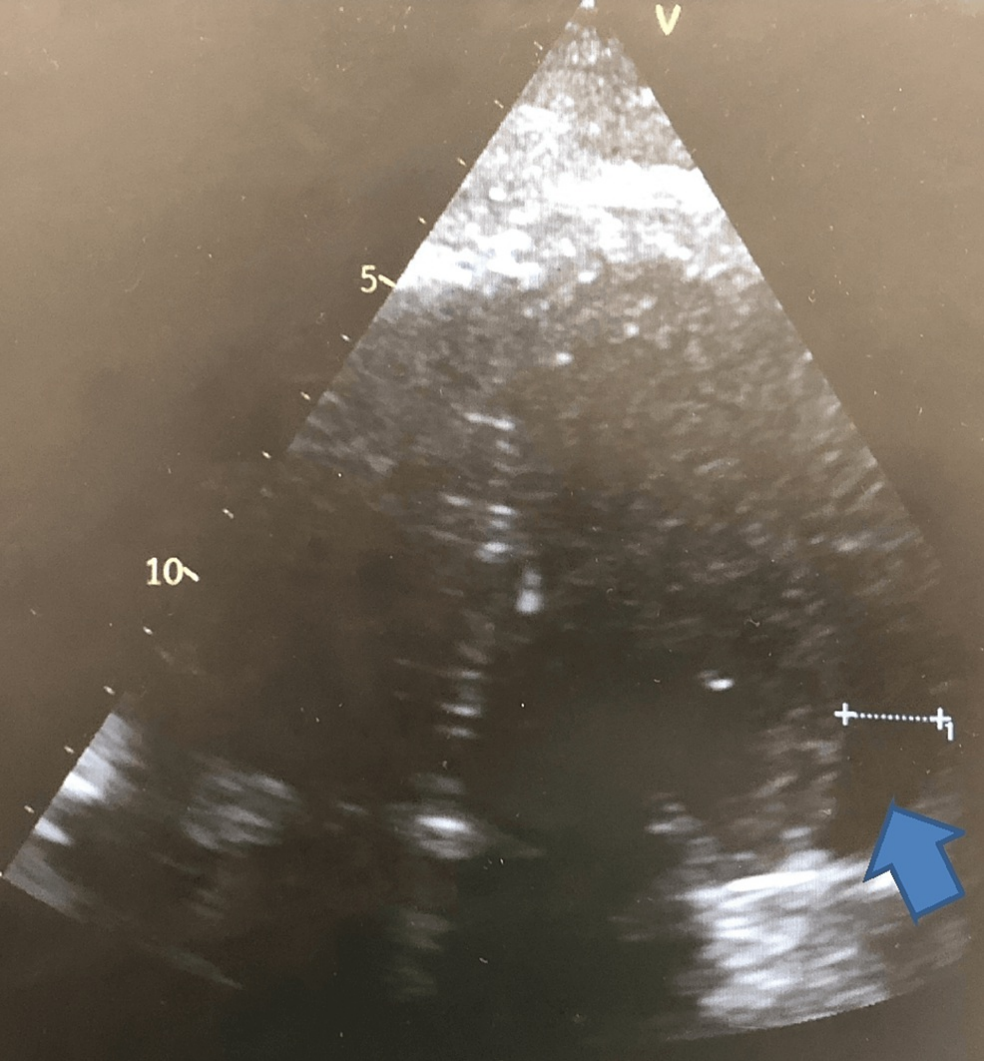
Apical 4 chamber echocardiographic view showing pericardial effusion (pointed blue arrow)

**Figure 2:**
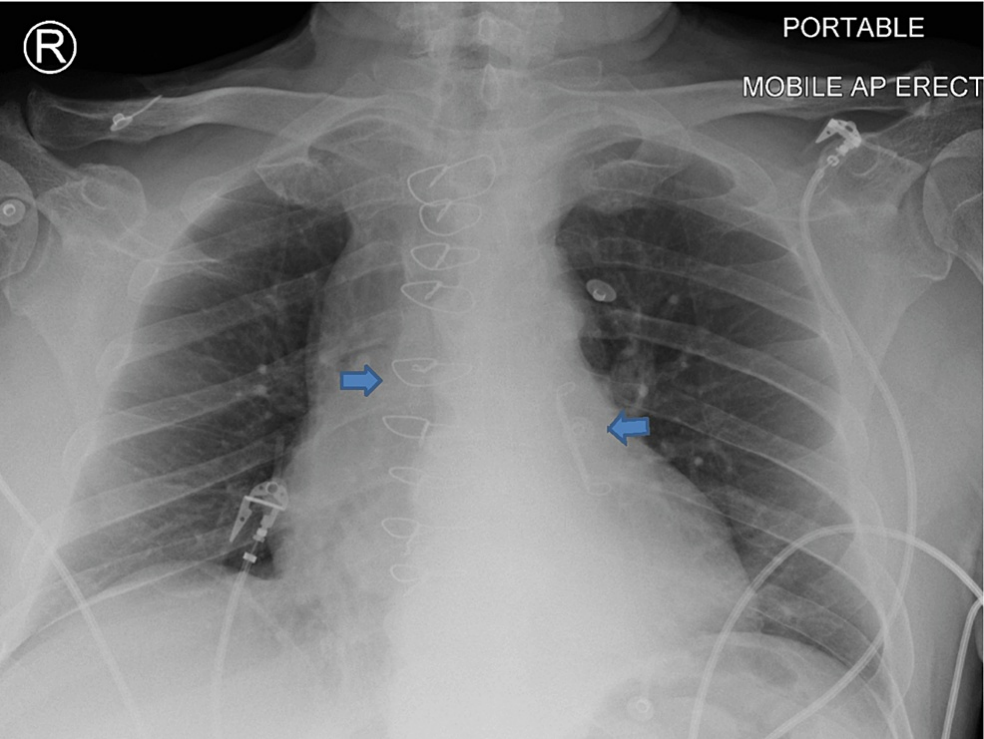
Antero-posterior chest radiography showing sternotomy sutures, and left atrial appendage closure device (pointed arrows).

A 70-year-old male known to have symptomatic paroxysmal AF with a previously performed failed DCCV and unresponsive to medications, presented for an elective AF pulmonary venous isolation (PVI) catheter ablation. His past medical history included hypertension, previous transient ischaemic attack, pre-diabetes, stage 2 chronic kidney disease with a baseline estimated glomerular filtration rate (eGFR) of 53, early Dupuytren's contracture, carpal tunnel syndrome, and previous radiotherapy for prostate cancer. The patient underwent pre-procedural TOE before the PVI that did not show any LAA thrombus or pericardial effusion. A TOE-guided trans-septal puncture was performed and left atrial (LA) 3D mapping was started. However, during the LA mapping, a significant drop in blood pressure was noted and hence emergency TOE images were obtained. A large pericardial effusion indicating tamponade was noted, and an emergency pericardial drain was inserted, which drained about 1 liter of hemorrhagic pericardial effusion. He received protamine and octaplex to reverse the effects of anticoagulation and to achieve hemostasis. The pericardial fluid reaccumulated quickly requiring an urgent sternotomy procedure and a large amount of blood including clots was evacuated by opening the pericardium achieving good output and the bleeding was repaired with two 4/0 pledgeted prolines. The LAA was identified to be the source of bleeding and a 35 mm atriclip was applied achieving hemodynamic stability. He underwent PVI twice during the surgery given hemodynamic stability and the patient returned to normal sinus rhythm post-PVI ablation. His blood pressure dropped during the procedure to 90 systolic, however, improved to 140/70 after the sternotomy and PVI ablation. Postoperatively, the patient maintained normal sinus rhythm and was admitted to the intensive care unit (ICU) for close monitoring. TTE performed two days later showed a moderate pericardial effusion infero-laterally and by the right atrium (RA) measuring laterally about 1.37 cm, basal posterior 0.9 cm, and 0.5 cm by the RA. TTE demonstrated preserved left ventricular function with ejection fraction > 55% and no obvious regional wall motion abnormalities (Videos 1, 2, Figure 1). Chest radiography showed sternotomy sutures, a left atrial appendage closure device, and a central venous cannulation line (Figure 2).

**Video 1:** Apical 4 chambers view showing pericardial effusion

**Video 2:** Apical two chambers echocardiographic view showing pericardial effusion

The pericardial drain was kept overnight and removed 24 hours later. Lab tests showed hemoglobin 105 g/L before surgery which dropped to 95 g/L post procedure. Creatinine was 76 µmol/L (normal range 59-104 µmol/L), C reactive protein 113 mg/L (normal range 0-5 mg/L), white cell counts 13.0 x10^9^/L (normal range 4-10), neutrophils 10.6 x10^9^/L (normal range 2-7), platelet count 560 x10^9^/L (normal range 150-410). He was stepped down from ICU the following day, and he initially had slightly low urine output that gradually improved. The patient was discharged home on oral bisoprolol and was also given a course of oral Co-Amoxiclav 625 mg three times daily for seven days due to small left basal changes on the chest radiography. The patient was discharged home four days later on bisoprolol 3.75 mg once in the morning and 2.5 mg once in the evening. Other medications included apixaban 5 m twice daily, dapagliflozin 10 mg once daily, lansoprazole 30 mg once daily, rosuvastatin 5 mg once daily, and tamsulosin MR 400 mg once daily. The patient had high CHADS-VASc score of 4 and anticoagulation was continued for the next two to three months. 

**Figure 3:**
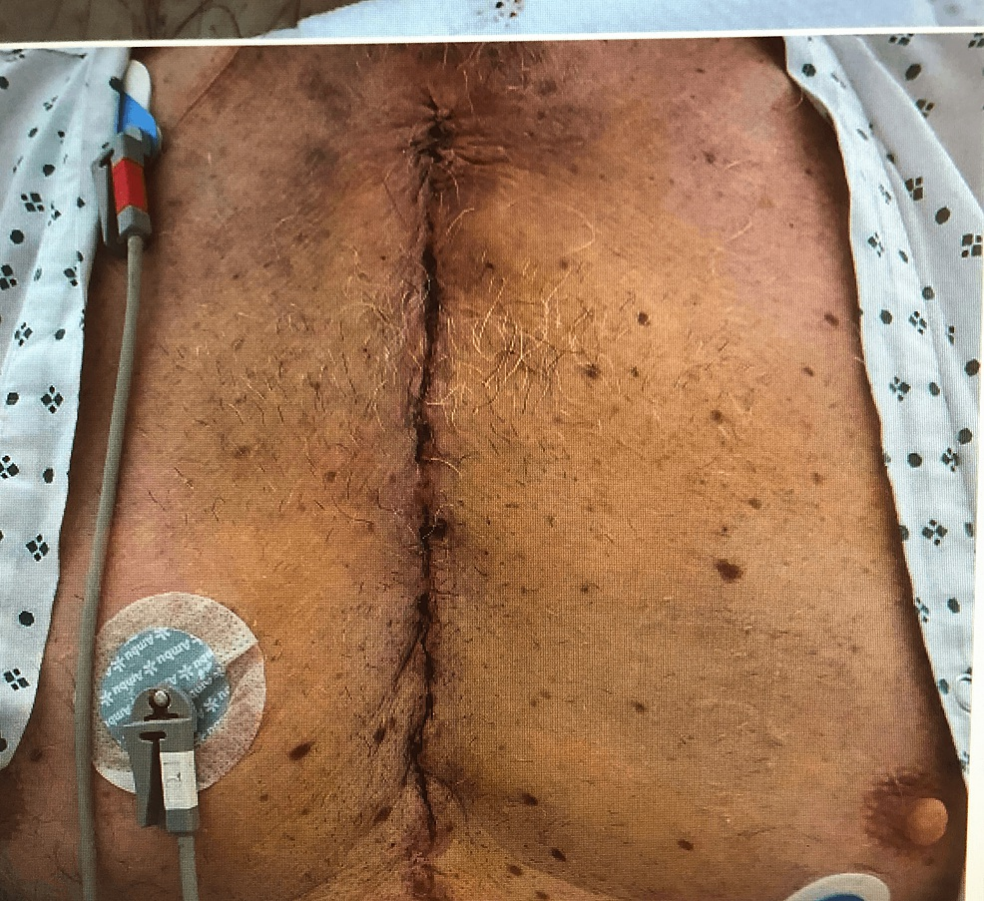
Sternotomy wound showing normal healing process without any signs of infection

He was readmitted to hospital a few days later following an episode of collapse and loss of consciousness. Emergency services ECG showed 2:1 fast atrial flutter with a heart rate of 146 bpm. Subsequently, bisoprolol was switched to sotalol 40 mg twice daily initially, which was then uptitrated to 80 mg twice daily to achieve rhythm control following a successful synchronized DCCV shock of 360 joules. The bedside echo did not show any reaccumulation of the pericardial effusion. The patient was discharged home with outpatient follow-up and has remained in normal sinus rhythm since. His sternotomy wound has healed without any complications (Figure 3).

## Discussion

AF is the most common clinically encountered symptomatic cardiac arrhythmia worldwide. The presenting clinical features vary considerably among patients from palpitations, chest pain, dyspnea, and dizziness, to potentially life-threatening conditions such as ischemic stroke which is a complication of AF [6]. Treatment of AF is complex and variable and depends on the intended objectives, as there are multiple modalities of treatments available, and often multiple modalities can be utilized simultaneously. Currently, available options are medical therapy, which can be medication strategies that result in rate control or pharmacological rhythm control in addition to medications to the prevention of complications such as stroke [5], electrical cardioversion cardiac electrophysiologic ablation procedures such as catheter and cryo-balloon ablations, a cardiac structural device to prevent strokes such as left atrial appendage (LAA) closure device and, in some cases, surgical interventions [7]. Elderly patients over 65 years old who have minimal symptoms are usually recommended to be treated with a rate control medication strategy as it has been demonstrated that this strategy results in improved symptoms and decreased hospitalizations and is associated with less side effect profile or complications [8,9].

Nonetheless, in severely symptomatic patients, young patients < 65 years old, or individuals with a first diagnosis of AF, the primary objective of AF treatment is to maintain a normal sinus rhythm [10]. The restoration of normal sinus rhythm results in the resolution of symptoms and therefore an improvement in the quality of life. The most anti-arrhythmic drugs utilized to chemically cardiovert AF are class Ic and IIIc anti-arrhythmic medications. However, those anti-arrhythmic are associated with significant side effect profiles and poor tolerance [7].

Another established strategy of rhythm control in symptomatic AF patients is catheter ablation which aims to isolate or exterminate foci that initiate and sustain the AF [10]. Catheter ablation is associated with higher success in eliminating AF than medical therapy [11] and is associated with comparable if not reduced adverse cardiovascular outcomes when compared to medical rhythm control [12]. Moreover, catheter ablation can be the first line of treatment in some patients [13]. However, catheter ablation is an invasive procedure that remains associated with considerable complications despite the reduction in the incipience of AF catheter-related complications over the year with a low incidence of overall complications of around 4.51% and severe complications of around 2.44% [10]. One of the uncommon yet potentially fatal is cardiac perforation, with both atrial and right ventricular apex being the most common site of perforations during ablations [14]. A perforation to the LAA seldom happens during AF ablation subsequently causing a tamponade similar to this case discussed. In two similar reported cases, surgical closure of the LAA by AtriClip was also used successfully [15]. This can be useful in preventing further AF-related complications.

The Japanese Registry of All Cardiac and Vascular Diseases‐Diagnosis Procedure Combination (JROAD‐DPC) study demonstrated that between April 2012 and March 2018 from 1,058 hospitals, 135,299 patients with AF underwent catheter ablation in 456 hospitals [16]. The study reported an in‐hospital complication rate of 3.4%. Cardiac tamponade incidence was reported as 1.2% and in‐hospital mortality was about 0.04% [16]. Certain factors such as old age, women, lower body mass index, and higher burden of comorbidities such as hypertension and diabetes were associated with higher complication risk in multivariate analysis. Cheng et al. reported a significant increase in quarterly rates of mortality and procedural complications following catheter ablation for AF between 2010 and 2015 which could be due to an increased number of comorbidities in these patients such as 26.9% of patients having ischaemic heart disease [17].

Yoshiaki et al. reported 29 events in 27 patients with cardiac tamponade who underwent catheter ablation for AF. 23 patients had structurally normal hearts, one had atrial septal defect closure, one had mild to moderate aortic regurgitation and two exhibited hypertrophic obstructive cardiomyopathy (HOCM) [3]. Cardiac tamponade was caused by intracardiac catheter manipulation in 24 patients, atrial septum puncture in three patients, and postprocedural inflammation in two patients, respectively. Twenty three of the 29 events occurred during the ablation procedure, four during ward stay and two events occurred during the first 33 days post procedure. Catheter ablation was completed in 19 events whereas in 10 events, the catheter ablation could not be completed on the first attempt. Cardiac tamponade was managed through percutaneous pericardial puncture in 21 events and seven events were managed conservatively whereas in one patient, a puncture was not attempted due to difficult anatomy [3,18].

Yoshimoto et al. reported that there is a lack of anticoagulation strategy in patients post AtriClip device procedure [19]. They recommended a three-month anticoagulation strategy post procedure to minimize risk of stroke or thromboembolism in patients. The stroke free rate during the follow-up for patients in this study was 98.9% despite having an estimated stroke risk of 4% based on the CHA2DS2-Vasc score for the entire cohort confirming the effectiveness of this strategy. Kim et al. reported in their study that patients continued with oral anticoagulation for 110 days and 90% of patients were able to discontinue the oral anticoagulant therapy which showed 73% reduction in risk of CHA2DS2-Vasc score predicted stroke [20]. The PROTECT AF trial demonstrate the efficacy of LAA exclusion in stroke prevention in patients not on anticoagulant therapy [21]. The expert consensus based on this study is not to use anticoagulant therapy in patients post AtriClip but instead to start aspirin on day 1 post-operatively and to continue indefinitely thereafter [22].

## Conclusions

AF is a very common disease with significant mortality and morbidity and catheter ablation is becoming a more common long-term management strategy to restore normal sinus rhythm. The procedure is associated with certain risks although the complications risk has reduced recently. Cardiac tamponade is a rare life-threatening complication of catheter ablation and patients usually require emergency intervention to avoid a fatal outcome. Patients may require percutaneous pericardial drain or sternotomy depending on the severity of the complication.

## References

[1] Parameswaran R, Al-Kaisey AM, Kalman JM (2021). Catheter ablation for atrial fibrillation: current indications and evolving technologies. Nat Rev Cardiol.

[2] Ishiguchi H, Yoshida M, Kawabata T, Yoshiga Y, Shimizu A, Oda T (2020). Delayed cardiac tamponade in catheter ablation for paroxysmal atrial fibrillation induced by a subacute hemorrhage. HeartRhythm Case Rep.

[3] Yui Y, Sekiguchi Y, Nogami A (2019). Midterm outcomes of catheter ablation for atrial fibrillation in patients with cardiac tamponade. J Arrhythm.

[4] Hsu LF, Jaïs P, Hocini M (2005). Incidence and prevention of cardiac tamponade complicating ablation for atrial fibrillation. Pacing Clin Electrophysiol.

[5] Gupta A, Perera T, Ganesan A (2013). Complications of catheter ablation of atrial fibrillation: a systematic review. Circ Arrhythm Electrophysiol.

[6] Hamaya R, Miyazaki S, Taniguchi H (2018). Management of cardiac tamponade in catheter ablation of atrial fibrillation: single-centre 15 year experience on 5222 procedures. Europace.

[7] Guan F, Stähli BE, Jakob P, Wolber T (2022). Perforation of multipolar electroanatomic mapping catheter in the left atrial appendage during left atrial mapping. HeartRhythm Case Rep.

[8] Li J, Gao M, Zhang M, Liu D, Li Z, Du J, Hou Y (2020). Treatment of atrial fibrillation: a comprehensive review and practice guide. Cardiovasc J Afr.

[9] Andrade JG (2017). My approach to atrial fibrillation: rate vs rhythm control. Trends Cardiovasc Med.

[10] Pellman J, Sheikh F (2015). Atrial fibrillation: mechanisms, therapeutics, and future directions. Compr Physiol.

[11] Prystowsky EN, Padanilam BJ, Fogel RI (2015). Treatment of atrial fibrillation. JAMA.

[12] Benali K, Khairy P, Hammache N (2023). Procedure-related complications of catheter ablation for atrial fibrillation. J Am Coll Cardiol.

[13] Asad ZU, Yousif A, Khan MS, Al-Khatib SM, Stavrakis S (2019). Catheter ablation versus medical therapy for atrial fibrillation: a systematic review and meta-analysis of randomized controlled trials. Circ Arrhythm Electrophysiol.

[14] Andrade JG, Wazni OM, Kuniss M (2021). Cryoballoon ablation as initial treatment for atrial fibrillation. J Am Coll Cardiol.

[15] Cappato R, Calkins H, Chen SA (2009). Prevalence and causes of fatal outcome in catheter ablation of atrial fibrillation. J Am Coll Cardiol.

[16] Yokoyama Y, Miyamoto K, Nakai M (2021). Complications associated with catheter ablation in patients with atrial fibrillation: a report from the Jroad-DPC study. J Am Heart Assoc.

[17] Cheng EP, Liu CF, Yeo I (2019). Risk of mortality following catheter ablation of atrial fibrillation. J Am Coll Cardiol.

[18] Rathore K, Saklani P, Ng J (2020). Surgical deployment of AtriClip in perforated left atrial appendage during catheter ablation. Ann Thorac Surg.

[19] Collado FM, Lama von Buchwald CM, Anderson CK (2021). Left atrial appendage occlusion for stroke prevention in nonvalvular atrial fibrillation. J Am Heart Assoc.

[20] Yoshimoto A, Suematsu Y, Kurahashi K, Kaneko H, Arima D, Nishi S (2021). Early and middle-term results and anticoagulation strategy after left atrial appendage exclusion using an epicardial clip device. Ann Thorac Cardiovasc Surg.

[21] Kim JY, Jeong DS, Park SJ, Park KM, Kim JS, On YK (2022). Long-term efficacy and anticoagulation strategy of left atrial appendage occlusion during total thoracoscopic ablation of atrial fibrillation to prevent ischemic stroke. Front Cardiovasc Med.

[22] Bedeir K, Warriner S, Kofsky E, Gullett C, Ramlawi B (2019). Left atrial appendage epicardial clip (AtriClip): essentials and post-procedure management. J Atr Fibrillation.

